# Activation of the apoptosis signal-regulating kinase 1/c-Jun N-terminal kinase pathway is involved in the casticin-induced apoptosis of colon cancer cells

**DOI:** 10.3892/etm.2014.1934

**Published:** 2014-08-26

**Authors:** LIN QU, FENG-XIA LIU, XIAO-CHENG CAO, QIAO XIAO, XIAOHONG YANG, KAI-QUN REN

**Affiliations:** 1 Department of Examination, The Third Xiangya Hospital of Central South University, Changsha, Hunan 410013, P.R. China; 2 Laboratory of Medicine, Medical College, Hunan Normal University, Changsha, Hunan 410016, P.R. China

**Keywords:** colon cancer, casticin, reactive oxygen species, apoptosis signal-regulating kinase 1, c-Jun N-terminal kinase, Bim, apoptosis

## Abstract

Casticin is one of the main components of the fruits of *Vitex rotundifolia* L. Studies have shown that casticin inhibits the growth of various cancer cells, including colon cancer. In the present study, the anti-carcinogenic effects of casticin on human colon cancer and the underlying mechanisms were investigated. The results revealed that casticin significantly induced apoptosis of HT-29, HCT-116, SW480 and Caco-2 cells, induced the accumulation of reactive oxygen species (ROS) and increased the protein levels of apoptosis signal-regulating kinase 1 (ASK1), c-Jun N-terminal kinase (JNK) and B-cell lymphoma 2-interacting mediator of cell death (Bim) in HT-29 cells. Pretreatment with N-acetylcysteine, an antioxidant chemical compound, inhibited the activation of ASK1, JNK and Bim, as well as the apoptosis induced by casticin. Small interfering RNA targeting ASK1 significantly attenuated the induction of JNK and Bim activation and apoptotic cell death by casticin treatment. SP600125, a specific JNK inhibitor, attenuated Bim activation and apoptosis, but did not alter ASK1 phosphorylation levels. In addition, casticin treatment resulted in apoptosis by the same mechanism in HCT-116, SW480 and Caco-2 cells. These results suggest that casticin significantly induced apoptosis by the activation of the ASK1-JNK-Bim signaling cascade and the accumulation of ROS in colon cancer cells.

## Introduction

Colorectal cancer (CRC) is one of the leading causes of cancer mortality in the world. The incidence rate of CRC has increased in a number of Asian countries, including China, during the past few decades (1,2). Currently, the prognosis for CRC remains poor, as little effective therapy has been developed (3). Therefore, developing effective therapeutic agents to treat CRC is an urgent requirement.

Casticin is a primary component of the fruits of *Vitex rotundifolia* L. that, for thousands of years, has been extensively used as an anti-inflammatory agent in Traditional Chinese Medicine (4). A number of studies have shown that casticin inhibits the growth of various cancer cells, including breast (5), lung (6) and colon cancer (7,8). In addition, our previous studies demonstrated that casticin induced apoptotic cell death of cervical cancer and hepatocellular carcinoma cells (9–11), even without functional p53. Therefore, investigations into the apoptosis-inducing effects and the underlying molecular mechanisms of casticin in p53-mutated human colon cancer cell lines are required.

The generation of reactive oxygen species (ROS) occurs in a number of biological systems, and ROS are well known to act as important determinants in the regulation of cell signaling pathways associated with proliferation, apoptosis and senescence (12). Other agents have also been found to generate ROS in the mitochondria, including diallyl trisulfide and several chemopreventive agents, such as benzyl isothiocyanate, phenethyl isothiocyanate and sulforaphane, by inhibiting complexes I or III of the mitochondrial respiratory chain and disrupting the mitochondrial membrane potential (13). During mitochondrial respiration, O_2_ acts as the terminal acceptor of electrons, with the four-electron reduction of O_2_ yielding H_2_O. In normal tissue, this has been estimated to occur 96–99% of the time. However, the one-electron reduction of O_2_ can yield superoxide; this is believed to occur in the electron transport chain at Sites I (nicotinamide adenine dinucleotide-dehydrogenase) or III (ubiquinone-cytochrome b) (14). Manganese superoxide dismutase catalyzes the dismutation of this superoxide to generate H_2_O_2_ and O_2_. The H_2_O_2_ is subsequently detoxified to H_2_O and O_2_, either through the action of glutathione (GSH) peroxidase in the mitochondria or, if it diffuses into the cytosol, by catalase in the peroxisomes. We previously reported that casticin induced apoptosis by causing ROS generation in cervical cancer cells (9,11); however, whether casticin stimulates ROS production in colon cancer cells is unclear.

Apoptosis signal-regulating kinase 1 (ASK1) is a multifunctional serine/threonine protein kinase that is involved in a wide range of physiological processes, including cell differentiation and apoptosis (15). ASK1 has been reported to be activated by a number of stress signals, including ROS, tumor necrosis factor-α and endoplasmic reticulum (ER) stress (16,17). B-cell lymphoma 2 (Bcl-2)-interacting mediator of cell death (Bim) is a member of the ‘BH3-only proteins’, a subgroup of Bcl-2 apoptotic regulators that contain only one of the Bcl-2 homologous regions (BH3). In response to apoptotic stimuli, BH3-only proteins undergo translocation from a number of cellular compartments to the mitochondrial membranes, where they interfere with the function of anti-apoptotic Bcl-2 family members, ultimately resulting in apoptotic cell death (18,19). In the oxidizing environment created by ROS, ASK1, an upstream protein in the c-Jun N-terminal kinase (JNK)-associated signal transduction pathway phosphorylation, causes activation of the JNK pathway (20). We previously showed that casticin induced apoptotic cell death of cervical cancer cells through the ROS-dependent activation of JNK (11). However, the role of the ASK1/JNK signaling cascade in casticin-induced colon cancer cell apoptosis remains unknown. In the present study, the anti-carcinogenic effects of casticin on human colon cancer were investigated.

## Materials and methods

### Chemicals and materials

Casticin was purchased from Biopurify Phytochemicals Ltd. (Chengdu, China). The compound has a molecular weight of 374.3, appears as yellow crystals and has a purity of 98.0%. Casticin was prepared in dimethyl sulfoxide (DMSO) as a 10 mmol/l stock solution and diluted in medium to the indicated concentration prior to use. 2′,7′-Dichlorofluorescein diacetate (DCFH-DA) was obtained from Molecular Probes (Eugene, OR, USA). Propidium iodide (PI), ethidium bromide, N-acetylcysteine (NAC, an oxygen-free radical scavenger) and SP600125 (a JNK inhibitor) were purchased from Sigma-Aldrich (St. Louis, MO, USA). Anti-JNK1 and β-actin antibodies were purchased from Santa Cruz Biotechnology, Inc. (Santa Cruz, CA, USA), and anti-phosphorylated JNK1/2, -phosphorylated ASK1, -ASK1, -phosphorylated Bim-extra long (EL) and -Bim-EL antibodies were purchased from Cell Signaling Technology, Inc. (Beverly, MA, USA).

### Cell lines and cell culture

The human colon cancer cell lines HT-29, HCT-116, SW480 and Caco-2 were purchased from the China Center for Type Culture Collection (Wuhan, China). The study was approved by the ethics committee of Hunan Normal University (Changsha, China). Cells were maintained in Dulbecco’s modified Eagle’s medium supplemented with 10% fetal bovine serum, 4 mM glutamine, 100 U/ml penicillin and 100 μg/ml streptomycin, and incubated at 37°C in a humidified atmosphere of 5% CO_2_.

### Flow cytometric analysis using PI staining

Cells were seeded at a density of 4×10^6^ cells/well in 250-ml culture flasks for 24 h and then treated with the medium containing various concentrations of casticin or 0.1% DMSO for 24 h, or 10.0 μM casticin for the indicated time periods. PI staining for DNA content analysis was performed as described previously (21). All analyses were performed using a flow cytometer (Coulter Epics XL-MSL, Beckman Coulter, Fullerton, CA, USA) with CellQuest™ software (BD Pharmingen, San Diego, CA, USA).

### Histone/DNA ELISA for the detection of apoptosis

The cell apoptosis ELISA detection kit (Roche, Basel, Switzerland) was used to detect apoptosis in cells treated with casticin according to the manufacturer’s instructions. Briefly, cells were seeded in a 96-well plate at a density of 1×10^4^ cells/well for 24 h, the tested agents were added and the cells were then cultured in RPMI-1640 medium containing 10% fetal bovine serum. After 24 h, the cytoplasm of the control and treatment groups was transferred to the 96-well plate peridiumed by the streptavidin and incubated with the biotinylated histone antibody and peroxidase-tagged mouse anti-human DNA (both from Roche, Palo Alto, CA, USA) for 2 h at room temperature. The absorbance at 405 nm was measured with an enzyme-linked immunosorbent apparatus (ELX-800 type; Bio-Tek, Shanghai, China).

### DNA agarose gel electrophoresis

Cells were seeded at a density of 4×10^6^ cells/well in 250-ml culture flasks for 24 h and treated with medium containing various concentrations of the test/control agents or vehicle and 10% fetal bovine serum for 24 h. This assay was performed as previously described (21).

### Measurement of ROS generation

ROS generation in the control and casticin-treated cells was measured by flow cytometry (FCM) following staining with the DCFH-DA. Briefly, cells were seeded in six-well plates (1×10^5^ cells per well), allowed to attach overnight and exposed to DMSO (control) or the desired concentrations of casticin for specified time periods. The cells were stained with 20 μM DCFH-DA for 30 min at 37°C, and the fluorescence intensity of the DCF in the cells was determined using the flow cytometer with winMDI software (Microsoft Corp., Redmond, WA, USA). As a rule, 10,000 cells were counted in each determination.

### Transfection of small interfering RNA (siRNA)

Control siRNA and siRNA targeting ASK1 were obtained from Santa Cruz Biotechnology, Inc. The sense sequences of the siRNA reagents were 5′-GACGCGATCAGAGAGTAAT-3′ (siRNA control) and 5′-GGTGGCACAAGCAAGTTCT-3′ (siRNA ASK1). For transient siRNA transfection, HT-29 cells were seeded at a density of 5×10^5^ cells/ml into six-well plates. Cells were transfected on the following day with the Lipofectamine^®^ LTX with Plus™ reagent (Invitrogen Life Technologies, Carlsbad, CA, USA) containing 100 ng/well siRNA (ASK1 or control). Cells were transfected with each siRNA and incubated for 48 h. The interference of ASK1 protein expression was confirmed by western blot analysis using the anti-ASK1 antibody.

### Western blot analysis

Western blot analysis was performed as described previously (22). Anti-JNK1, -phosphorylated JNK1/2, -phosphorylated ASK1, -ASK1, -phosphorylated Bim-EL, -Bim-EL and -β-actin antibodies were used as the primary antibodies. The signals were detected using an ECL Advance western blot analysis system (Amersham Pharmacia Biotech, Inc., Piscataway, NJ, USA).

### Statistical analysis

Data are presented as the mean ± standard deviation for triplicate experiments and were analyzed using the Student’s t-test. Differences from the controls were considered significant when P<0.05.

## Results

### Casticin induces apoptosis in colon cancer cells

**Figure 1:**
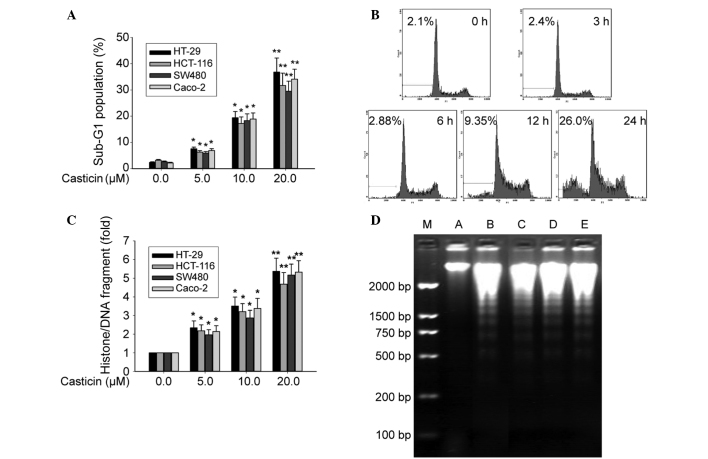
Casticin induces apoptosis of colon cancer cells. (A) Human colon cancer cells were treated with the indicated concentrations of casticin for 24 h. Data are presented as the mean ± standard deviation (n=3). ^*^P<0.05 and ^**^P<0.01 vs. 0 μM casticin. (B) HT-29 cells were treated with 10 μM casticin for the indicated time period. The DNA contents of the cells were analyzed by flow cytometry. (C) The cells were treated with the indicated concentrations of casticin for 24 h. The apoptotic effects of casticin were detected by the cell apoptosis ELISA detection kit. Data are presented as the mean ± standard deviation (n=3). ^*^P<0.05 and ^**^P<0.01 vs. 0 μM casticin. (D) The apoptotic effects of casticin were detected by DNA agarose gel electrophoresis in colon cancer cells. Lanes: M, DNA marker (DL2000); A, HT-29 cells treated with 0 μM casticin; B, HT-29 cells treated with 10.0 μmol/l casticin; C, HCT-116 cells treated with 10.0 μmol/l casticin; D, SW480 cells treated with 10.0 μmol/l casticin; E, Caco-2 cells treated with 10.0 μmol/l casticin. All treatments were for 24 h.

It has been previously reported that casticin significantly inhibits the proliferation of human colon cancer cells (7,8). To further investigate its mechanisms, the hypodiploid cell populations were detected by FCM. Fig. 1A shows that casticin increased the percentage of the sub-G1 population in a concentration-dependent manner both in the p53 mutant cell line HT-29, and in the HCT116, SW480 and Caco-2 cell lines (P<0.05). The sub-G1 population of HT-29 cells treated with casticin was increased at 12 h and peaked at 24 h (Fig. 1B). The histone/DNA fragments of the HT-29, HCT-116, SW480 and Caco-2 cells, as measured by the cell apoptosis ELISA detection kit, were increased in a dose-dependent manner (P<0.05) following treatment with casticin (Fig. 1C). Furthermore, DNA fragmentation analysis by agarose gel electrophoresis revealed a typical ladder pattern of internucleosomal DNA fragments in the colon cancer cells that were treated with 10.0 μmol/l casticin for 24 h (Fig. 1D). These results suggest that casticin inhibits colon cancer cell proliferation by a mechanism involving the induction of apoptosis. The sequential experiments in the study explored the molecular mechanism by which casticin caused apoptosis using the p53-mutated human colon cancer HT-29 cells.

### Generation of ROS during treatment with casticin

**Figure 2:**
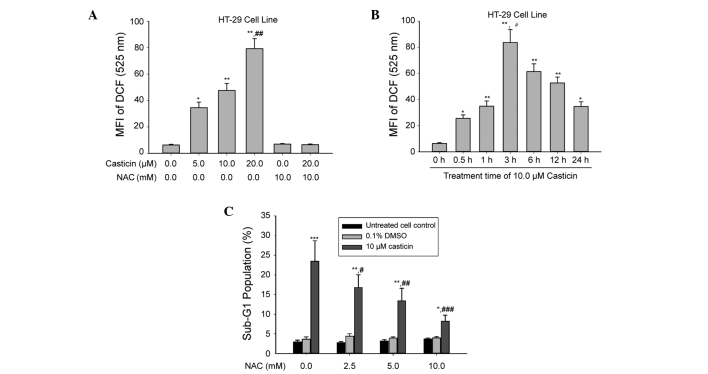
Casticin increases intracellular reactive oxygen species in HT-29 cells. (A) HT-29 cells were treated with the indicated concentrations of casticin for 3 h or pretreated with 10 mM NAC for 1 h followed by exposure to 20 μM casticin for 3 h. (B) HT-29 cells were treated with 10.0 μM casticin for the indicated time period. The MFI of DCF was measured by FCM using a 2′,7′-DCF diacetate fluorescence probe. (C) HT-29 cells were treated with 10.0 μM casticin for 24 h in the presence or absence of the indicated concentrations of 1 h NAC treatment. The DNA contents of the cells were analyzed by FCM. Data are presented as the mean ± standard deviation (n=3). ^*^P<0.05 and ^**^P<0.01 vs. 0 μM casticin or 0 h; ^#^P<0.05, ^##^P<0.01 and ^###^P<0.001 vs. the same concentration of casticin in combination with the indicated concentrations of NAC. MFI, mean fluorescence intensity; DCF, dichlorofluorescein; NAC, N-acetylcysteine; DMSO, dimethyl sulfoxide; FCM, flow cytometry.

We have previously demonstrated that the casticin-induced apoptosis of human cervical cancer cells is associated with the induction of ROS generation (9,11). Therefore, the present study investigated whether casticin also caused ROS production in colon cancer cells. Following the treatment of HT-29 cells with 5.0, 10.0 and 20.0 μM casticin for 3 h, the levels of ROS increased in a dose-dependent manner (Fig. 2A). Time-course experiments revealed that the levels of ROS increased initially at 0.5 h, reached a peak at 3 h and persisted for up to 24 h after treatment with 10.0 μM casticin (Fig. 2B).

To explore the role of ROS in casticin-induced colon cancer cell apoptosis, the antioxidant NAC was used. As shown in Fig. 2C, pretreatment of HT-29 cells with NAC (2.5, 5.0 and 10.0 mM) markedly attenuated casticin-induced apoptosis in a concentration-dependent manner. Furthermore, casticin-induced ROS production was almost completely inhibited by treatment with 10.0 mM NAC (Fig. 2A). These results suggest that casticin causes intracellular ROS generation, and the oxidative stress further contributed to apoptosis of the human colon cancer HT-29 cells.

### Activation of the JNK signal transduction pathway during treatment with casticin

**Figure 3:**
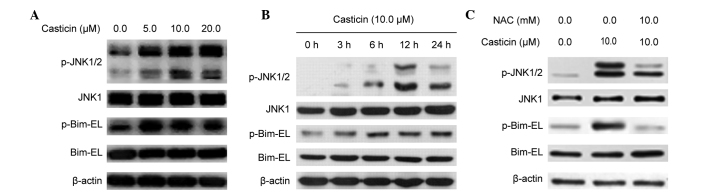
Casticin activates the JNK signal transduction pathway in HT-29 cells. **(**A) HT-29 cells were treated with the indicated concentrations of casticin for 12 h. (B) HT-29 cells were treated with 10.0 μM casticin for the indicated time period. (C) HT-29 cells were treated with 10.0 μM casticin for 12 h in the presence or absence of 10 mM NAC. The expression levels of p-JNK1/2, JNK1, p-Bim-EL and Bim-EL in total cellular extracts were determined by western blotting, and β-actin was used as the loading control. NAC, N-acetylcysteine; p-, phosphorylated-; JNK, c-Jun N-terminal kinase; Bim, Bcl2-interacting mediator of cell death; Bim-EL, Bim-extra long.

Our previous report indicated that ROS generation and sustained JNK activation play a role in the casticin-induced apoptosis of human cervical cancer cells (11). The present study examined whether casticin activates the JNK pathway in human colon cancer cells. Fig. 3A and B shows that casticin treatment increased the phosphorylation levels of the JNK1/2 protein and its downstream molecule Bim-EL, and these phosphorylation levels were attenuated in the presence of 10.0 mM NAC (Fig. 3C) in the HT-29 cells. These results suggest that casticin caused JNK activation through intracellular ROS generation in the colon cancer HT-29 cells.

### Activation of ASK1 during treatment with casticin

**Figure 4:**
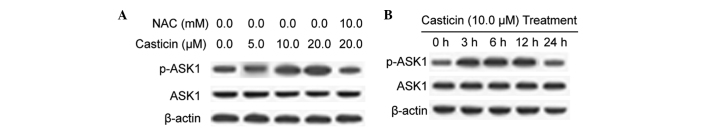
Casticin increases the level of ASK1 phosphorylation in HT-29 cells. (A) HT-29 cells were treated with the indicated concentrations of casticin for 12 h. (B) HT-29 cells were treated with 10.0 μM casticin for the indicated time period. The expression levels of p-ASK1 and total ASK1 in total cellular extracts were determined by western blotting, and β-actin was used as the loading control. NAC, N-acetylcysteine; p-, phosphorylated-; ASK1, apoptosis signal-regulating kinase 1.

ASK1 is a member of the mitogen-activated protein kinase kinase kinase (MAPKK) family that activates the JNK pathways by directly phosphorylating and thereby activating its respective MAPKKs, mitogen-activated protein kinase kinase (MKK) 4/7 and MKK3/6 (20). In the present study, we next examined whether the casticin-induced ROS activated ASK1. ASK1 is one of the upstream regulators of JNK, and is known to be associated with cell death (21,23). Fig. 4A and B shows that casticin induced the phosphorylation of ASK1 in a concentration- and time-dependent manner, and this was attenuated by pretreatment with 10 mM NAC in the HT-29 cells. These results suggest that, in the colon cancer HT-29 cells, the activation of ASK1 caused by casticin was dependent on intracellular ROS generation.

### Knockdown of ASK1 by siRNA inhibits casticin-induced JNK phosphorylation and apoptosis

**Figure 5:**
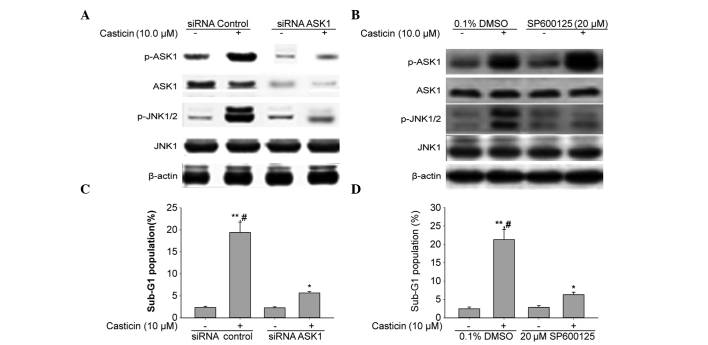
Downregulation of ASK1 using siRNA inhibits JNK1/2 activation and apoptosis by casticin in HT-29 cells. **(**A) HT-29 cells were transfected with 100 nM siRNA control or the siRNA duplexes against ASK1 mRNA. Forty-eight hours after the transfection, the cells were treated with 10 μM casticin for 12 h. (B) HT-29 cells were treated with 10 μM casticin for 12 h in the presence or absence of 20 μM SP600125. (A and B) To assess whether ASK1 activation was negatively regulated by JNK, western blotting of p-ASK1, ASK1, p-JNK1/2 and JNK1 was completed following the downregulation of ASK1 by (A) siRNA transfection and/or (B) the JNK inhibitor. β-actin was used as the loading control. (C) HT-29 cells were transfected with 100 nM of siRNA control or the siRNA duplexes against ASK1 mRNA. Forty-eight hours after the transfection, the cells were treated with 10 μM casticin for 24 h. ^*^P<0.05 and ^**^P<0.01 vs. siRNA control. (D) HT-29 cells were treated with 10 μM casticin for 24 h in the presence or absence of 20 μM SP600125. (C and D) The DNA contents of the cells were analyzed by flow cytometry. Data are presented as the mean ± standard deviation (n=3). ^*^P<0.05 and ^**^P<0.01 vs. 0.1% DMSO; ^#^P<0.05 and ^##^P<0.01 vs. the same concentration of casticin in combination with siRNA ASK1 transfection or 20 μM SP600125 treatment. siRNA, small interfering RNA; DMSO, dimethyl sulfoxide; ASK1, apoptosis signal-regulating kinase 1; p-, phosphorylated-; JNK, c-Jun N-terminal kinase.

To investigate the role of ASK1 in regulating JNK and inducing apoptosis caused by casticin, siRNA that specifically targeted ASK1 was used. The results of the western blot analysis showed that ASK1 was downregulated following the transfection of ASK1 siRNA in HT-29 cells (Fig. 5A). The effect of ASK1 siRNA on the activation of JNK caused by casticin treatment was examined. Fig. 5A shows that ASK1 siRNA attenuated the increased phosphorylation levels of JNK1/2 that were induced by casticin, suggesting that ASK1 mediates casticin-induced JNK activation.

In order to assess whether ASK1 activation is negatively regulated by JNK, the cells were treated with a pharmacological compound named SP600125, a known JNK inhibitor. The results from the western blot analysis showed that activated ASK1 was not altered by pretreatment with SP600125 (Fig. 5B). These results suggest that ASK1 was activated prior to JNK in HT-29 cells treated with casticin. Fig. 5C and D shows that the ASK1 siRNA and JNK inhibitor decreased the casticin-induced apoptosis in the HT-29 cells. We can conclude that casticin may have caused apoptosis by activating the ASK1-JNK signaling pathway in the HT-29 cells.

### Effects of casticin on the generation of ROS and phosphorylation of ASK1 and JNK in other human colon cancer cells

**Figure 6:**
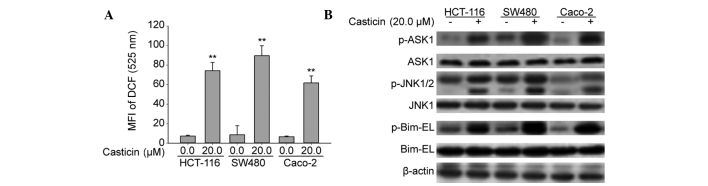
Casticin promotes the production of ROS and activation of ASK1, JNK and Bim in HCT-116, SW480 and Caco-2 cells. **(**A) Treatment of HCT-116, SW480 and Caco-2 cells with 20.0 μM casticin increased the intracellular ROS levels, as determined by flow cytometry using a 2′,7′-DCF diacetate fluorescence probe. ^**^P<0.01 vs. 0 μM casticin. (B) Treatment of HCT-116, SW480 and Caco-2 cells with 20.0 μM casticin increased the phosphorylation levels of ASK1, JNK1/2 and Bim-EL. β-actin was used as the loading control. MFI, mean fluorescence intensity; DCF, dichlorofluorescein; p-. phosphorylated-; ASK1, apoptosis signal-regulating kinase 1; JNK. c-Jun N-terminal kinase; Bim, Bcl2-interacting mediator of cell death; Bim-EL, Bim-extra long; ROS, reactive oxygen species.

We next investigated whether casticin induces apoptosis by the same modality in other colon cancer cell lines, including HCT-116, SW480 and Caco-2. As shown in Fig. 6A, FCM results obtained by monitoring the DCFH-DA probe indicated that casticin significantly induced ROS generation. Western blot analysis showed that casticin treatment also caused an increase in ASK1, JNK and Bim-EL phosphorylation levels in the HCT-116, SW480 and Caco-2 cells (Fig. 6B). Together, these findings suggest that casticin-induced apoptotic cell death, ROS generation and the activation of ASK1, JNK and Bim were not specific to human colon cancer cell types.

## Discussion

The present study examined the apoptotic mechanism of a potential chemopreventive agent, casticin, which is an active ingredient of Fructus Viticis that has been widely used as an anti-inflammatory drug in Traditional Chinese Medicine for thousands of years (4). Casticin significantly induced the apoptotic cell death of the human colon cell lines HT-29, HCT-116, SW480 and Cacao-2 and has been shown to exhibit similar effects on other cancer cells, including those of the breast (5), prostate (24), cervix (9,11), lung (6), liver (10) and hematological system (25). These observations suggest that casticin can be used as a chemopreventive agent for a variety of cancers.

Our present and previous studies have demonstrated that casticin functions as a chemopreventive agent by inducing apoptosis of tumor cells through the generation of ROS, which creates oxidative stress (Figs. 1 and 2) (9,11,21,26). We previously reported that the casticin-induced apoptosis of hepatocellular carcinoma cells is involved in GSH depletion (10). In present study, the results showed that pretreatment of HT-29 cells with NAC markedly attenuated casticin-induced apoptosis in a concentration-dependent manner. Furthermore, casticin-induced ROS production was almost completely inhibited by treatment with 10.0 mM NAC (Fig. 2). Therefore, it is possible that, due to the disruption of mitochondrial electron transport chain activity and/or the downstream GSH peroxidase/reductase system, casticin elevated the intracellular level of ROS in colon cancer cells; however, this mechanism requires further investigation in future studies.

In the present study, it was also revealed that casticin treatment activates the ASK1/JNK-associated signal transduction pathway (Figs. 3 and 4) by ROS production. ASK1 is activated by a variety of stresses, including calcium influx, ER stress, lipopolysaccharide, ROS and tumor necrosis factor (27). These stresses induce the activation of ASK1 by protein phosphorylation (28). A previous study revealed that the activation of ASK1 plays a central role in a wide range of cellular responses, including cell differentiation, apoptosis and the immune response, with a particular focus on oxidative stress-induced apoptosis (29). The present results show that casticin treatment induced ASK1 phosphorylation, which increased ASK1 activity. These findings suggest that casticin-induced apoptosis mainly occurs through ASK1 phosphorylation. The activation of ASK1 can selectively activate JNK, leading to apoptosis. The present study revealed that casticin treatment caused JNK phosphorylation. To confirm that ASK1 regulates JNK, siRNA was utilized to specifically downregulate ASK1 expression. The phosphorylation of JNK by casticin stimulation was revealed to be attenuated by siRNA targeting ASK1. However, the inhibition of JNK with the pharmacological inhibitor SP600125 had no effect on the phosphorylation of ASK1 protein, possibly indicating that ASK1 cannot be negatively regulated by JNK.

Bcl-2 family proteins regulate mitochondria-dependent apoptosis, with the balance of the anti- and proapoptotic members regulating rates of cell survival and death. Bim, a proapoptotic member of the Bcl-2 family, causes apoptosis by disrupting mitochondrial integrity (30). *Bim* gene expression occurs in response to selected stress signals. The present study revealed that casticin induced Bim-EL phosphorylation, suggesting that Bim-EL phosphorylation is causally associated with casticin-induced HT-29 cell apoptosis. Furthermore, casticin-induced Bim phosphorylation was inhibited by an antioxidant, the JNK inhibitor, and siRNA targeting ASK1. Thus, it is plausible that casticin activates the ROS-ASK1-JNK cascade causing Bim phosphorylation and subsequent apoptotic cell death. A previous study indicated that, in addition to Bim, other BH3-only members of the Bcl-2 family, including Bcl-2-associated agonist of cell death and Bcl-2-associated X protein, are involved in casticin-induced cancer cell apoptosis (31). These observations explain, at least in part, why blocking the ASK1 signaling cascade did not completely eliminate the casticin-induced HT-29 cell apoptosis. In conclusion, the present study demonstrates the possibility that the apoptotic mechanism of casticin is effected by the production of ROS, which causes activation of the ASK1-JNK-Bim pathway. The model used in the present study can also be used in future investigations of these associations.
